# Supplementary material for: Differentiation between Fresh and Thawed Cephalopods Using NIR Spectroscopy and Multivariate Data Analysis
Source: Foods. 2021 Mar 3;10(3):528. doi: 10.3390/foods10030528 (PMC7999131; doi:10.3390/foods10030528)
Supplement: Supplementary file 1 [file foods-10-00528-s001.pdf]

foods

Supplementary Materials

Differentiation between Fresh and Thawed Cephalopods Using NIR Spectroscopy and Multivariate Data Analysis

Francesco Pennisi [1,#], Alessandro Giraudo [2,#], Nicola Cavallini [2], Giovanna Esposito [1], Gabriele Merlo [3], Francesco Geobaldo [2], Pier Luigi Acutis [1], Marzia Pezzolato [1,*], Francesco Savorani [2,◆] and Elena Bozzetta [1,◆]

[1] Istituto Zooprofilattico Sperimentale del Piemonte, Liguria e Valle d'Aosta, Via Bologna 148, 10154 Turin, ITALY, francesco.pennisi@izsto.it (F.P.); giovanna.esposito@izsto.it (G.E.); pierluigi.acutis@izsto.it (P.L.A.); elena.bozzetta@izsto.it (E.B.);
[2] Department of Applied Science and Technology, Politecnico di Torino, Corso Duca degli Abruzzi 24, 10129 Turin, ITALY, alessandro.giraudo@polito.it (A.G.); nicola.cavallini@polito.it (N.C.); francesco.geobaldo@polito.it (F.G.); francesco.savorani@polito.it (F.S.);
[3] Esselunga S.p.A, Via Giambologna 1, 20096 Limito di Pioltello (MI), ITALY, gabriele.merlo@esselunga.it
[#] The authors equally contributed to this work
◆ The authors equally contributed to this work
* Correspondence: marzia.pezzolato@izsto.it; Tel: +39-011-2686-254

The Table of Contents:

Figure 1S. Example of raw NIR spectra of cuttlefish and musky octopus samples acquired using SCiO, MicroNIR, and MPA instruments.

Figure 2S. PCA loadings corresponding to the principal components shown in Figure 1.

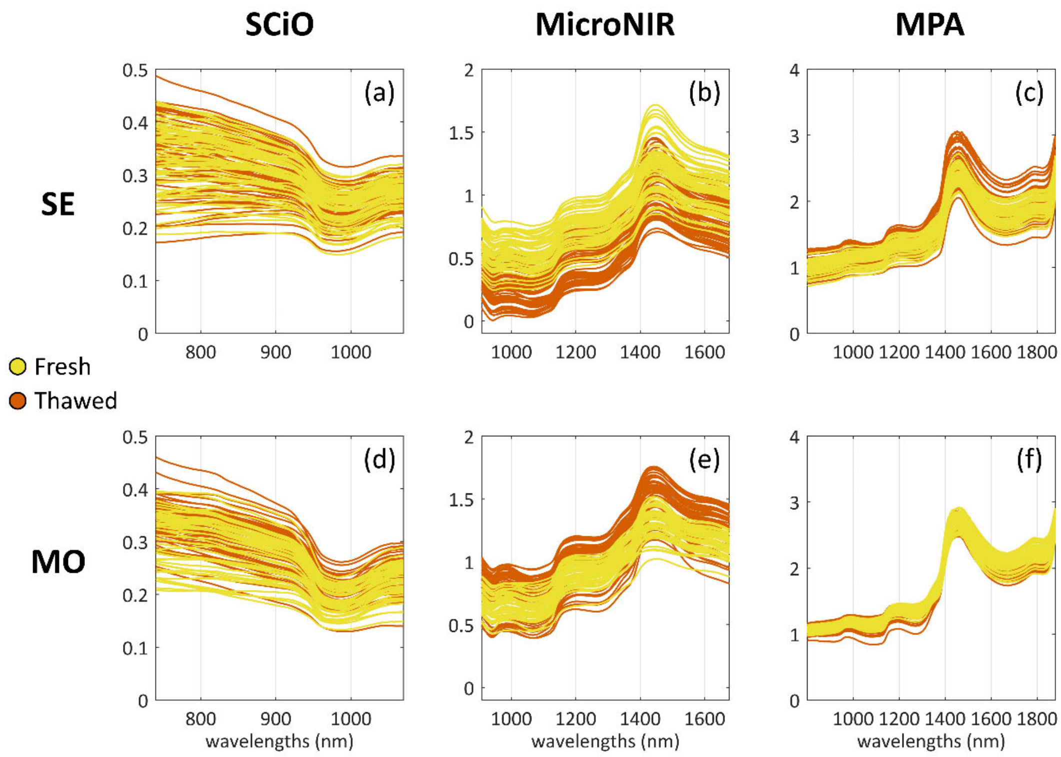

Figure 1S. Example of raw NIR spectra of cuttlefish ("SE"; a, b, c) and musky octopus ("MO"; d, e, f) samples acquired using SCiO, MicroNIR, and MPA instruments. Fresh samples are depicted in yellow, while thawed samples are colored in orange.

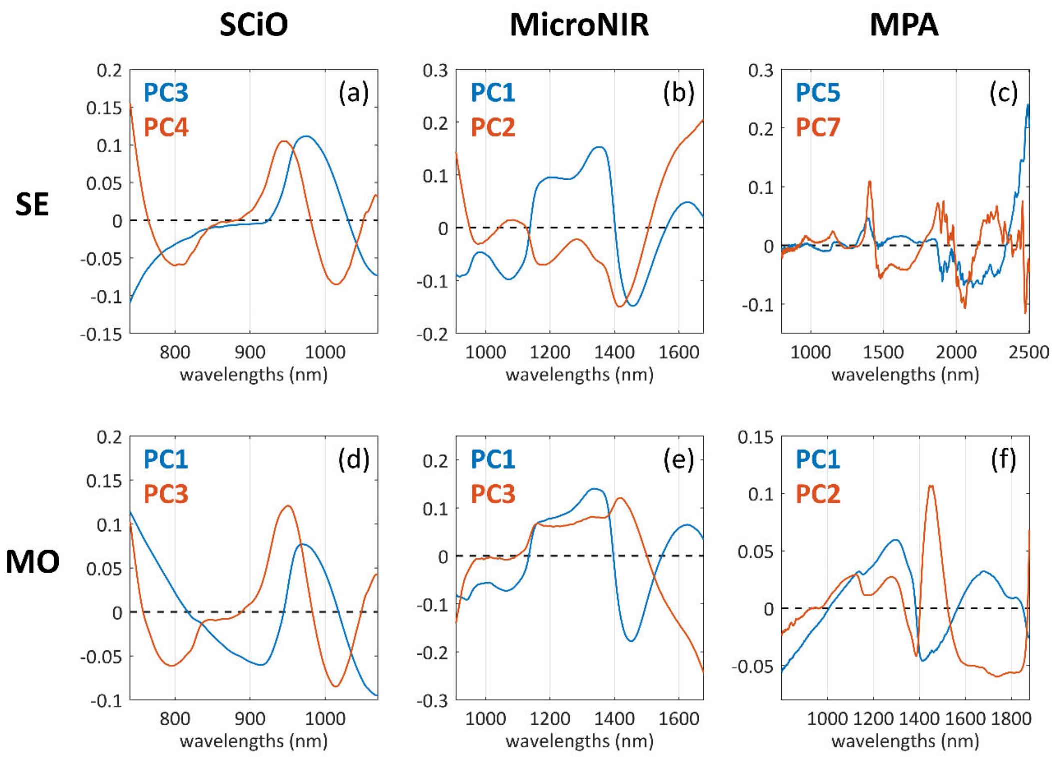

Figure 2S. PCA loadings corresponding to the principal components (PC) shown in Figure 1 (PCA score plots). Models corresponding to cuttlefish (“SE”; a, b, c) and musky octopus (“MO”; d, e, f) individual datasets are reported.
